# Patient-reported outcome measures (PROMs) after laparoscopic cholecystectomy: systematic review

**DOI:** 10.1093/bjsopen/zrac062

**Published:** 2022-06-07

**Authors:** Conor Melly, Gearoid McGeehan, Niall O’Connor, Alison Johnston, Gary Bass, Shahin Mohseni, Claire Donohoe, Magda Bucholc, Michael Sugrue

**Affiliations:** Department of Surgery, Letterkenny University Hospital and Donegal Clinical Research Academy, Donegal, Ireland; University of Limerick School of Medicine, University of Limerick, Limerick, Ireland; Department of Surgery, Letterkenny University Hospital and Donegal Clinical Research Academy, Donegal, Ireland; University of Limerick School of Medicine, University of Limerick, Limerick, Ireland; Department of Surgery, Letterkenny University Hospital and Donegal Clinical Research Academy, Donegal, Ireland; Department of Surgery, Letterkenny University Hospital and Donegal Clinical Research Academy, Donegal, Ireland; Division of Traumatology, Emergency Surgery and Surgical Critical Care, University of Pennsylvania, Philadelphia, Pennsylvania, USA; School of Medical Sciences, Orebro University, Orebro, Sweden; Division of Trauma and Emergency Surgery, Department of Surgery Orebro University Hospital, & School of Medical Sciences, Orebro University, Orebro, Sweden; Department of Surgery, Trinity College Dublin, St James’ Hospital, Dublin, Ireland; Intelligent Systems Research Centre, School of Computing, Engineering and Intelligent Systems, Ulster University, Magee Campus, Derry-Londonderry, UK; Department of Surgery, Letterkenny University Hospital and Donegal Clinical Research Academy, Donegal, Ireland; EU INTERREG Centre for Personalized Medicine, Intelligent Systems Research Centre, School of Computing, Engineering and Intelligent Systems, Ulster University, Magee Campus, Derry-Londonderry, UK

## Abstract

**Background:**

Healthcare requires patient feedback to improve outcomes and experience. This study undertook a systematic review of the depth, variability, and digital suitability of current patient-reported outcome measures (PROMs) in patients undergoing laparoscopic cholecystectomy.

**Methods:**

A PROSPERO-registered (registration number CRD42021261707) systematic review was undertaken for all relevant English language articles using PubMed version of MEDLINE, Scopus, and Web of Science electronic databases in June 2021. The search used Boolean operators and wildcards and included the keywords: laparoscopic cholecystectomy AND patient outcome OR patient-reported outcome OR patient-reported outcome measure OR PRO OR PROM. Medical Subjects Heading terms were used to search PubMed and Scopus. Articles published from 1 January 2011 to 2 June 2021 were included.

**Results:**

A total of 4960 individual articles were reviewed in this study, of which 44 were found to evaluate PROMs in patients undergoing laparoscopic cholecystectomy and underwent methodological index for non-randomized studies (MINORS) grading. Twenty-one articles spanning 19 countries and four continents met all inclusion criteria and were included in the qualitative data synthesis. There was significant heterogeneity in PROMs identified with eight different comprehensive PROM tools used in the 21 studies. There was wide variation in the time points at which PROMs were recorded. Fourteen of 21 studies recorded PROMs before and after surgery, and 7 of 21 recorded PROMs only after surgery. Follow-up intervals ranged from 3 days to 2 years after surgery.

**Conclusions:**

This study identified that while post-laparoscopic cholecystectomy PROMs are infrequently measured currently, tools are widely available to achieve this in clinical practice. PROMs may not capture all the outcomes but should be incorporated into future cholecystectomy outcome research. The EQ-5D™ (EuroQoL Group, Rotterdam, the Netherlands) provides a simple platform for the modern digital era.

## Teaser

This study undertook a PROSPERO-registered systematic review of current patient-reported outcome measures (PROMs) in laparoscopic cholecystectomy (LC) with the aim of making a recommendation on the most useful PROM in LC. Significant heterogeneity in PROM tools was identified with wide variations in the time points at which outcomes were recorded. We identified that EuroQoL Group EQ-5D™ provides a simple platform for the modern digital era.

## Introduction

Laparoscopic cholecystectomy (LC) is one of the most common operative procedures undertaken worldwide with approximately 18 million cholecystectomies performed annually^1–3^. Cholecystitis and cholecystectomy are associated with morbidity and occasional mortality^4^, particularly when performed in the emergency setting^5,6^. Most publications reporting outcomes relate to duration of hospital stay and early follow-up, often with a strong surgical focus and little information on long-term outcomes of the patients’ own experience.

Understanding patient outcomes through patient-reported outcome measures (PROMs) beyond the perioperative and early postoperative interval are key in the delivery of value-based healthcare. Studies suggest that surgeon experience, and long-term quality of life (QoL) are key influencers in favourable patient outcomes undergoing elective LC^7,8^. Other important factors are shared decision-making, communication, skill of the surgeon, and nursing care^8^. Of least importance to patients was day-case surgery, and scar cosmesis^8^. Parkin suggested that there is a disconnect between patients and surgeons regarding what constitutes important outcomes^7^. The National Institute for Health and Care Excellence (NICE) recommend greater incorporation of PROMs to report outcomes of importance to patients, including the continuation of symptoms and the onset of new symptoms that affect long-term QoL in patients undergoing LC^9^.

It is increasingly recognized internationally that there has been a lack of evaluation of PROMs and their experiences^10,11^. Outcome evaluation needs ideally to be a validated process, incorporating both the patient’s functional and QoL metrics as well as medical or process outcomes. To ensure the patient’s own total care experience is evaluated with PROMs, the PROM tool should capture the patient’s opinion of their health status and the benefits they have experienced from accessing health services^12^.

Existing studies utilizing targeted PROMs following cholecystectomy have enhanced our understanding of specific post-cholecystectomy symptoms of pain, diarrhoea, and wound-related complications^13^.

Many surgeons support the use of PROMs as an adjunct to improving clinical management, to elicit sensitive information and aid in patient counselling^14^. Furthermore, PROMs can influence the development of health policy and resource allocation^15^. Despite support for PROMs, they have not gained widespread traction in patients undergoing LC^8^. There is a lack of research on long-term outcomes and the impact of LC on patient outcomes. Many patients report the continuance of symptoms after LC, or the appearance of new symptoms. Research is required to establish the long-term benefits and harms, so that appropriate information may be provided to patients to aid in their decision-making and long-term management^9^.

The primary aim of this systematic review was to identify PROMs used to describe patient outcomes in LC. The secondary aim was to report on ease of use and functionality of PROMs and make a recommendation on the most useful PROMs in LC.

## Methods

### Search strategy

A systematic review was undertaken for all relevant English language articles using PubMed version of MEDLINE, Scopus, and Web of Science electronic databases in June 2021. The search used Boolean operators and wildcards and included the keywords: laparoscopic cholecystectomy AND patient outcome OR patient-reported outcome OR patient-reported outcome measure OR PRO OR PROM. Medical Subjects Heading terms were used to search PubMed and Scopus. Articles published from 1 January 2011 to 2 June 2021 were chosen to capture the current literature.

### Inclusion and exclusion criteria

The methods of the analysis and inclusion criteria were specified in advance to avoid selection bias and documented in a protocol registered with the PROSPERO database (www.crd.york.ac.uk/prospero) (registration number CRD42021261707). This systematic review adhered to PRISMA guidelines. Only studies reporting comprehensive PROM tools in patients undergoing LC that were full-text articles in the English language were included. Studies were not included if they were systematic reviews or meta-analyses or were designed as case reports, editorial comments, or letters; had fewer than 20 patients; contained paediatric populations; contained pregnant populations; or were transvaginal studies, as these PROMs focused on gynaecological symptoms. Citations were extracted onto Microsoft^®^ Excel and duplicates were removed.

### Study selection and data extraction

Studies identified by the search strategy were screened for inclusion initially by title, then abstract, and subsequently by full-text review. Eligibility assessment was performed by two independent reviewers (C.M. and G.M.G.). Disagreements were resolved by consensus and if no agreement could be reached, a third reviewer (A.J.) decided.

Three reviewers (C.M., G.M.C., and N.O.C.) independently assessed each published study for the quality of study design and risk of bias by way of standardized pre-piloted forms, calculating a methodological index for non-randomized studies (MINORS) score. A MINORS score of 18 or greater out of 24 for comparative studies and 12 or greater out of 16 for non-comparative studies was considered the standard for inclusion^16^.

A standardized data sheet was created, and data were collected on the details of publication, study design, number of patients, patient characteristics (mean age and sex), emergency or elective operation, and PROM-specific details (patient-reported outcomes (PROs), instruments used to assess PROs, survey distribution, response rates, and follow-ups). Where a study reported PROMs from both a laparoscopic approach and open procedures, or those initiated as laparoscopic procedures converted to open, only the data pertaining to the laparoscopic approach was used.

An assessment of bias was performed with the Cochrane risk of bias tool for the following domains: random sequence generation; allocation concealment; blinding of participants and personnel; blinding of outcome assessment; incomplete outcome data; selective reporting bias; and other bias^17^.

## Results

**Fig. 1:**
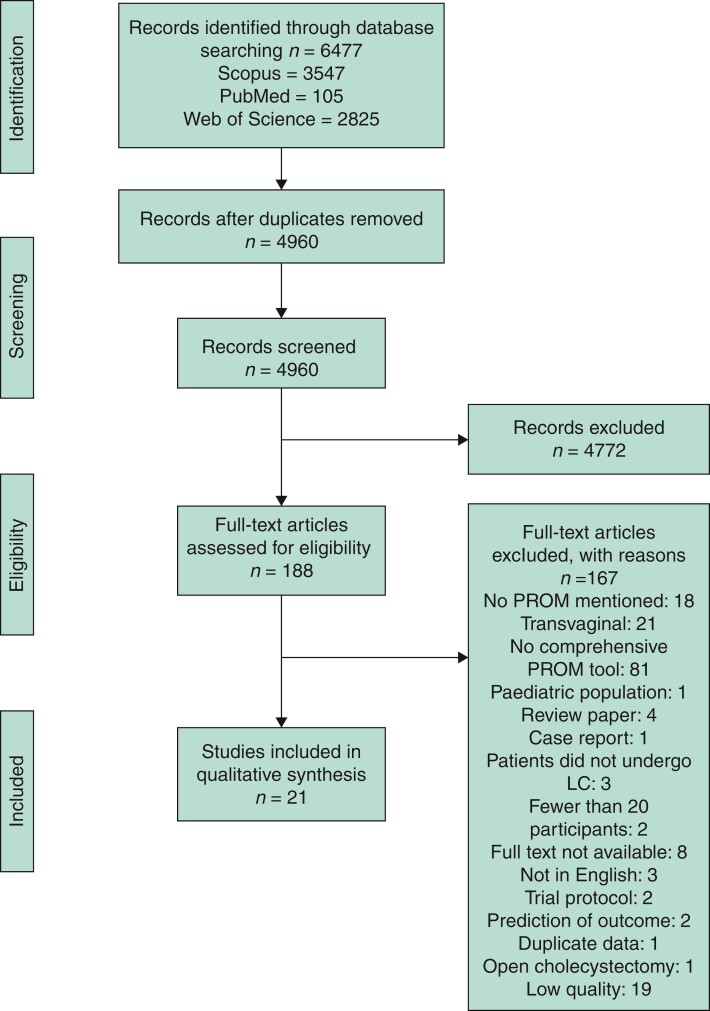
Identification, review, and selection of articles included in systematic review

PROM, patient-reported outcome measure; LC, Laparoscopic cholecystectomy.

**Fig. 2:**
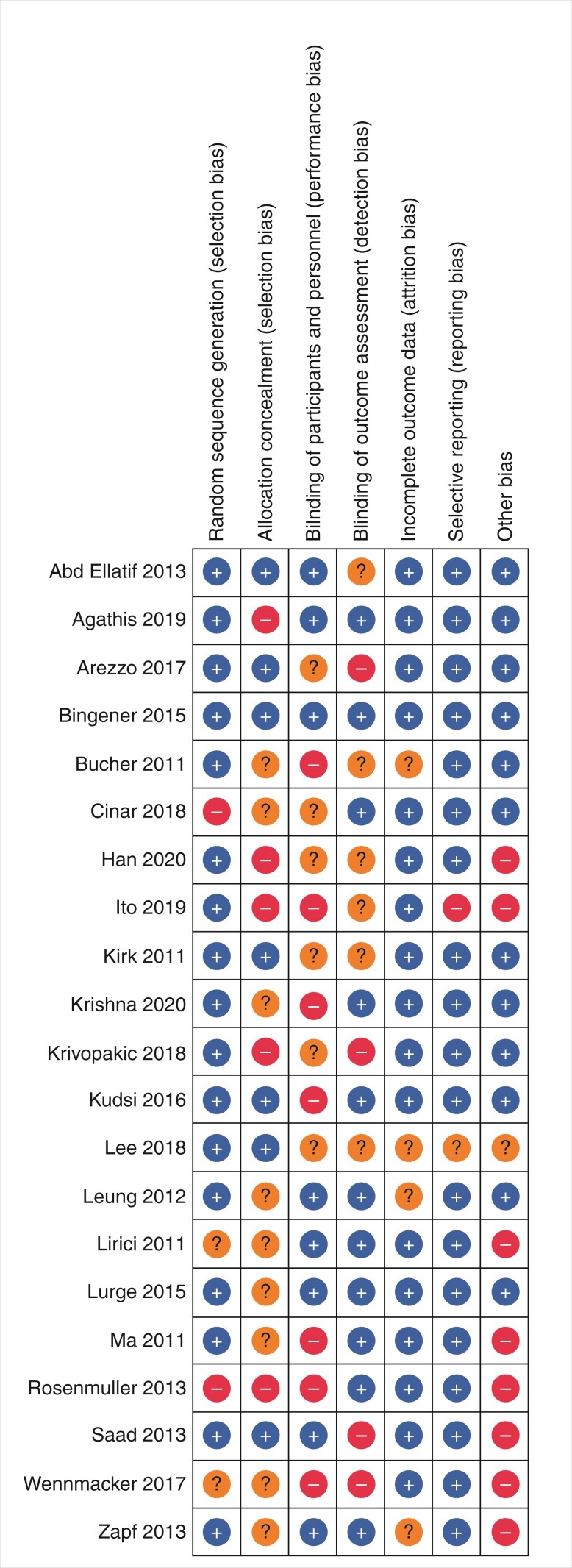
Risk of bias summary: review authors’ judgements about each risk of bias item for each included study

A total of 4960 individual articles were reviewed in this study (*Fig. 1*) of which 44 were found to be relevant and underwent MINORS grading. Twenty-one articles spanning 19 countries and four continents met all inclusion criteria and were included in the qualitative data synthesis. Of these, 12 of 21 were randomized clinical trials (RCTs), 7 were prospective studies, and 2 were retrospective. Eleven studies used multicentre databases. *Table 1* shows the characteristics of the studies included in this review. Results from the Cochrane risk of bias assessment are represented in *Fig. 2*.

**Table 1:** Study characteristics based on patient-reported outcome measure used

Author	Year	Country	Study design	No. of centres	Sample size	Intervention	Control	PROM used
**Han**	2020	South Korea	Pro	5	476	4-port LC	None	GIQLI
**Krishna**	2020	India	RCT	1	94	SILS	4-port LC	WHOQoL-BREF
**Ito**	2019	Japan	RCT	3	117	SILS	4-Port LC	SF-36^®^ subsets
**Agathis**	2019	USA	Pro	1	30	4-port LC	None	GI/SF-12 subsets
**Lee**	2018	Taiwan	Pro	2	672	4-Port LC	None	SF-36^®^, GIQLI
**Cinar**	2018	Turkey	Retro	2	142	SILS	4-Port LC	SF-36^®^
**Krivopakic**	2018	Serbia	Pro	1	40	4-Port LC	None	WHOQoL-BREF
**Arezzo**	2017	Multiple	RCT	20	600	SILS	4-port LC	GIQLI
**Wennmacker**	2017	Netherlands	Retro	1	146	4-port LC	None	GIQLI
**Kudsi**	2016	Multiple	RCT	8	136	Robotic SILS	4-port LC	SF-12
**Lurge**	2015	Switzerland	RCT	2	96	SILS	4-port LC	SF-36^®^
**Bingener**	2015	USA	RCT	1	110	SILS	4-port LC	PROMIS-10
**Saad**	2013	Germany	RCT	1	105	SILS, MiniLC	4-port LC	GIQLI
**Zapf**	2013	USA	Pro	1	100	SILS	4-port LC	SOMS
**Rosenmuller**	2013	Sweden	RCT	2	333	Open	4-port LC	EQ-VAS (EuroQoL)
**Abd Ellatif**	2013	Egypt	RCT	1	250	SILS	4-port LC	EuroQoL EQ-5D™
**Leung**	2012	USA	RCT	3	79	SILS	4-port LC	SOMS
**Kirk**	2011	UK	Pro	N/R	158	4-port LC	None	GIQLI
**Lirici**	2011	Italy	Pro	2	40	SILS	4-port LC	SF-36^®^
**Ma**	2011	USA	RCT	1	43	SILS	4-port LC	SF-36^®^
**Bucher**	2011	Switzerland	RCT	1	150	SILS	4-port LC	SF-12

PROM, patient-reported outcome measure; Pro, prospective study; Retro, retrospective study; RCT, randomized clinical trial; LC, laparoscopic cholecystectomy; SILS, single incision laparoscopic cholecystectomy; GIQLI, gastrointestinal quality of life index; WHOQoL-BREF, world health organization qulity of life; SF-36, short form-36; GI/SF-12, gastrointestinal/short form-12; PROMIS-10, patient reported outcomes measurement information system; SOMS, surgical outcomes measurement system; EQ-VAS, euro quality of life-visual analogue scale; EQ-5D, euro quality of life-5D.

A total of 3917 patients underwent LC and completed PROMs. One study did not report on mean age and sex of the 600 patients included in their study. One study did not report on the sex of the 150 patients included in their study. The mean age of the 3317 patients with available age data was 49.5 years. Of the 3167 patients with data on sex, 64 per cent were female and 36 per cent were male.

There was significant variation in the PROM tools used. A total of eight different comprehensive PROM tools were used, including: gastrointestinal QoL index (GIQLI)^18–23^, Short Form 36 (SF-36^®^, RAND Corporation, Santa Monica, CA, USA)^19,24–28^, SF-12^29–31^, World Health Organization QoL (WHOQoL)-BREF^32,33^, surgical outcomes measurement system (SOMS)^34,35^, euro quality of life (EQ-VAS)^36,37^, and PRO measurement information system (PROMIS)-10^38^. The most frequently used PROMs were the GIQLI (6 of 21) and the SF-36^®^ (6 of 21).

The use of selective PROMs was identified for pain (19 of 21), cosmesis (8 of 21), overall satisfaction (2 of 21), productivity loss and sick leave (1 of 21), and healthcare consumption (1 of 21). Two-thirds of studies measured PROMs before and after surgery, with a significant variation in follow-up intervals ranging from 3 days up to 2 years as shown in *Table 2*. *Table 2* also represents the number of patients lost to follow-up. Studies with a shorter follow-up interval reported fewer patients lost to follow-up, whereas Kirk and colleagues had a 2-year follow-up interval and reported that 22 per cent of patients missed at least one follow-up^23^. Nine studies reported no loss to follow-up, and six studies excluded any patients that were lost to follow-up from their analysis. Two studies did not report patients lost to follow-up. In 18 studies, the PROMs were completed by the patients alone, in 1 study the patients completed the PROM along with a nurse^18^, and in 1 study the patients completed the PROM along with a dedicated research coordinator^34^. One study did not report who completed the PROM^19^.

**Table 2:** Time points at which patient-reported outcome measures were administered and description of patients lost to follow-up

Author	Time points	Loss to follow-up with reason
**Han**	1 and 12 months after surgery	20 refused survey by telephone at 12 months. Excluded from analysis
**Lee**	Before and 2 years after surgery	14 lost (unknown reason), 11 refused to participate. Excluded from analysis
**Arezzo**	60 days after surgery	59 patients refused to participate or there were missing data. Excluded from analysis
**Wennmacker**	Before and 24 weeks after surgery	No loss to follow-up
**Saad**	10 days after surgery	2 lost to follow-up. Excluded from analysis
**Kirk**	Before, 6 weeks, 3 months, 2 years after surgery	35 (22.2 per cent) failed to complete at least one questionnaire after surgery
**Cinar**	Various times after surgery	5 lost to follow-up – unable to communicate
**Ito**	Before, daily until 14–20 days after surgery	1 withdrew, 5 failed to return questionnaire. Excluded from analysis
**Lurge**	12 weeks and 1 year after surgery	7 withdrew. Excluded from analysis
**Lirici**	1 month after surgery	No loss to follow-up
**Ma**	Various times after surgery	No loss to follow-up
**Agathis**	Before and after surgery	No loss to follow-up
**Kudsi**	Before, 2 weeks, 6 weeks, 3 months after surgery	7 lost at 2 weeks, 24 lost at 6 weeks, 22 lost at 3 months. No reason given
**Bucher**	Before, 30 days after surgery	No loss to follow-up
**Krishna**	Before, 3 months after surgery	No loss to follow-up
**Krivopakic**	Before, 1 day, 2 days, 3 days after surgery	No loss to follow-up
**Zapf**	Before, 24 h, 72 h, 1 week, 3 months, 6 m, 1 year, 2 years after surgery	Not reported
**Leung**	2 years after surgery	Not reported
**Rosenmuller**	3 days, 7 days, 11 days, 30 days after surgery	No loss to follow-up
**Abd Ellatif**	Before, 1 week, 1 month, 6 months after surgery	No loss to follow-up
**Bingener**	Before, 4 h, 1 day, 7 days after surgery	2 did not return calls or mail

Fourteen of the 21 studies reported that the PROM tool was administered as a questionnaire, and 7 studies did not comment on mode of administration of the PROM tool. Of these 14 studies, the PROM tool was distributed at the clinic (3 of 14), at the clinic and over the telephone (2 of 14), over the telephone and by mail (1 of 14), at the clinic and by mail (1 of 14), at the clinic and by mail or e-mail depending on patient preference (1 of 14), and by mail (1 of 14). Five of the 14 studies that administered the PROM as a questionnaire did not report on how they distributed the questionnaire.

The time taken to complete the PROM was reported in 2 of the 21 studies^25,28^. This was between 15 and 20 min per patient to complete the SF-36^®^, visual analogue scale pain score, and patient overall, and cosmetic satisfaction on a 10-point scale. It took 5 min per patient to complete two subscales of the SF-36^®^: the role physical subscale and the bodily pain subscale. None of the 21 studies reported on the cost associated with the use of a PROM tool.

None of the studies stated that they used a digital platform, and none of the studies mentioned the need for ethical approval.

*Table 3* provides a description of the domains measured by each of the PROM tools such as mobility, self-care, usual activities, pain/discomfort, and anxiety/depression measured by the EQ-5D™. Domains measured by the GIQLI include gastrointestinal symptoms, physical status, emotional status, and social function status, whereas the PROMIS-10 measures overall physical health, mental health, social health, pain, fatigue, and overall perceived QoL.

**Table 3:** Description of the parameters measured by the patient-reported outcome measures

PROM	Areas measured	References
**GIQLI**	GIQLI is a 36-question survey, with five response levels to each survey question. It records the health status of a patient and responses as ‘all the time, most of the time, some of the time, a little of the time, or never’. The data ate four subgroups: gastrointestinal symptoms (19 questions, total score 0–76), physical status (7 questions, total score 0–28), emotional status (5 questions, total score 0–20), and social function status (5 questions, total score 0–20).	^18–23^
**SF-36^®^**	It comprises 36 questions that cover eight domains of health: Limitations in physical activities because of health problems. Limitations in social activities because of physical or emotional problems Limitations in usual role activities because of physical health problems Bodily pain General mental health (psychological distress and wellbeing) Limitations in usual role activities because of emotional problems Vitality (energy and fatigue) General health perceptions	^19,24–28^
**SF-12**	Shortened version of the SF-36^®^, it uses the same eight domains as the SF-36^®^	^29–31^
**PROMIS-10**	The PROMIS Global-10 short form has 10 items that assess general domains of health and functioning including overall physical health, mental health, social health, pain, fatigue, and overall perceived quality of life.	^38^
**SOMS**	SOMS outcomes include physical function, impact of pain on quality of life, cosmesis, fatigue, bowel function, and overall satisfaction with results.	^34,35^
**WHOQoL-BREF**	The WHOQoL-BREF is a self-administered questionnaire comprising 26 questions on the individual’s perceptions of their health and wellbeing. Responses to questions are on a 1-5 Likert scale where 1 represents ‘disagree’ or ‘not at all’ and 5 represents ‘completely agree’ or ‘extremely’. The WHOQoL-BREF covers four domains each with specific facets: Physical health Activities of daily living Dependence on medicinal substances and medical aids; energy and fatigue Mobility; pain and discomfort Sleep and rest; work capacity Psychological Bodily image and appearance Negative feelings; positive feelings; self-esteem Spirituality/religion/personal beliefs Thinking, learning, memory, and concentration Social relationships Personal relationships; social support; sexual activity Environment Financial resources; freedom, physical safety, and security Health and social care: accessibility and quality; home environment Opportunities for acquiring new information and skills Participation in and opportunities for recreation/leisure activities Physical environment (pollution/noise/traffic/climate); transport There are also two separate questions which ask specifically about the individual’s overall perception of their health; and the individual’s overall perception of their quality of life.	^32,33^
**EQ-VAS**	The EQ-VAS records patient’s self-rated health on a vertical VAS, from ‘best health you can imagine’ to worst health. The VAS acts as a quantitative measure of health outcome for patient’s own judgement. The descriptive system has five dimensions: mobility, self-care, usual activities, pain/discomfort, and anxiety/depression.	^36,37^
**EQ-5D™**	Measures patient health across five different domains: mobility, self-care, usual activities, pain/discomfort, and anxiety/depression. Each dimension has five levels: no problems, slight problems, moderate problems, severe problems, and extreme problems.	^36,37^

PROM, patient-reported outcome measure; SF-36^®^, Short Form 36; SF-12, short form-12; SOMS, surgical outcomes measurement system; WHOQoL-BREF, World Health Organization quality of life-BREF; GIQLI, gastrointestinal quality of life index; PROMIS-10, Patient reported outcome measurement information system 10; EQ, euro quality of life; VAS, visual analogue scale.

*Table 4* provides a representation of the author’s reason for their choice of PROM tool in each study. Four authors chose a PROM because it was validated in their language, and seven selected a tool because it has been validated previously. Three studies chose the GIQLI as it had been previously validated in patients undergoing LC or gallbladder surgery^18,19,22^.

**Table 4:** Reasons given by authors for their choice of patient-reported outcome measure tool

Author	PROM	Reason for PROM choice
**Han**	GIQLI	Widely used for QoL in gallbladder surgery, recommended by European Association for Endoscopic Surgery
**Lee**	GIQLI/SF-36^®^	SF-36^®^ validated in Chinese, GIQLI validated, and reliable in patients undergoing cholecystectomy. Chinese version validated
**Arezzo**	GIQLI	Not reported
**Wennmacker**	GIQLI	Validated in Dutch (patients with potentially operable periampullary carcinoma)
**Saad**	GIQLI	Developed, validated, and tested in patients undergoing LC
**Kirk**	GIQLI	Extensively validated, sufficiently responsive to detect changes in health status after surgery for asymptomatic gallstones
**Cinar**	SF-36^®^	Validation performed in Turkish
**Ito**	SF-36^®^	Validated measurement tool, role physical subscale detected a greater difference in QoL in LC patients than other subscales
**Lurge**	SF-36^®^	The most accepted and validated health profile is the SF-36^®^
**Lirici**	SF-36^®^	Not reported
**Ma**	SF-36^®^	Allows comparisons of burden of illness among diseases and populations, equally applicable to all persons; regardless of condition
**Agathis**	GI/SF-12	GI survey developed as an abbreviated questionnaire modelled after the GIQLI. SF-12
**Kudsi**	SF-12	Measures perceived health and describes physical health status and mental health distress
**Bucher**	SF-12	Not reported
**Krishna**	WHOQoL-BREF	Paucity of data comparing QoL in 4-port LC and SILS. GIQLI previously used and did not detect a difference in the 2 groups of interest
**Krivopakic**	WHOQoL-BREF	Serbian translation available and validated
**Zapf**	SOMS	SOMS is an extension of the NIH funded PROMIS
**Leung**	SOMS	Validated questionnaire
**Rosenmuller**	EQ-VAS	Not reported
**Abd Ellatif**	EQ-5D™	Validated questionnaire. Simple instrument used to measure health outcomes. previous studies did not detect a difference in SF-36^®^ scores
**Bingener**	PROMIS-10	PROMIS items are more sensitive to change than other tools such as SF-36^®^. Used previously and found to be responsive to changes in patients after laparoscopic surgery

PROM, patient-reported outcome measure; SF-36^®^, Short Form 36; SF-12, short form-12; WHOQoL-BREF, World Health Organization quality of life-BREF; GIQLI, gastrointestinal quality of life index; PROMIS-10, patient reported outcomes measurement information system 10; SOMS, surgical outcomes measurement system; EQ, euro quality of life; VAS, visual analogue scale; LC, laparoscopic cholecystectomy; SILS, single incision laparoscopic surgery.

It seems that all PROM tools had the ability to detect changes in QoL measures at different follow-up intervals. These results are summarized in *Table 5*. It is unclear from analysis of the studies included in this systematic review whether the PROM tools were able to adequately capture resolution of symptoms, onset of new symptoms, or persistence of symptoms following LC as this was not reported or analysed by the authors. A summary of the features of each PROM tool used is shown in *Table 6*.

**Table 5:** Representation of the ability of patient-reported outcome measure tools to detect changes in quality of life over time

Author	PROM	Improvement in QoL
**Han**	GIQLI	QoL improved at 12 months compared with 1 month after surgery, no baseline data recorded
**Lee**	SF-36^®^, GIQLI	Both GIQLI and SF-36^®^ measured improvements in QoL at 2 years after surgery compared with preoperative baseline
**Arezzo**	GIQLI	QoL recorded at 1 time point only
**Wennmacker**	GIQLI	Not reported
**Saad**	GIQLI	QoL recorded at 1 time point only
**Kirk**	GIQLI	QoL improved at 6 weeks, 3 months, 2 years after surgery compared with baseline
**Cinar**	SF-36^®^	QoL recorded at one time point only
**Ito**	SF-36^®^	Not reported
**Lurge**	SF-36^®^	Measured time to return to preoperative baseline only
**Lirici**	SF-36^®^	QoL recorded at one time point only
**Ma**	SF-36^®^	Not reported
**Agathis**	SF-12 subsets	QoL improved at postoperative follow-ups compared with before surgery
**Kudsi**	SF-12	QoL returned to preoperative baseline within 3 months after surgery
**Bucher**	SF-12	QoL improved at 30 days after surgery compared with before
**Krishna**	WHOQoL-BREF	Not reported
**Krivopakic**	WHOQoL-BREF	QoL returned to pre-op baseline at day 2 after surgery and improved from preoperative baseline on day 3 after surgery
**Zapf**	SOMS	QoL improved over the follow-up interval
**Leung**	SOMS	QoL returned to preoperative baseline and began to improve in the follow-up interval
**Rosenmuller**	EQ-VAS	No baseline recorded, QoL scores improved over the postoperative interval
**Abd Ellatif**	EQ-5D	QoL improved from preoperative baseline
**Bingener**	PROMIS-10	QoL returned to baseline by 1 week after surgery

PROM, patient-reported outcome measure; QoL, quality of life; SF-36^®^, Short Form 36; SF-12, short form-12; WHOQoL-BREF, World Health Organization quality of life-BREF; GIQLI, gastrointestinal quality of life index; PROMIS-10, Patient reported outcomes measurement information system 10; SOMS, surgical outcomes measurement system; EQ, euro quality of life; VAS, visual analogue scale.

**Table 6:** Summary of the features of each patient-reported outcome measure tool

PROM	No. of items	Scoring	Time to complete	Availability	Languages	Assesses diarrhoea	Validated	Cost
**EQ-5D**	5 questions with 5 options	Quick and easy	1 min	Laptop, paper, tablet, phone app	200	No	Yes	Free
**WHOQoL-BREF**	26 questions with 5 options	Guide online, time consuming	N/A	Paper only	19	No	Yes	Free
**SF-36^®^**	36 questions with multiple options	Scoring systems available, time consuming	15–20 min	Paper only	Multiple, with translation guidelines available	No	Yes	Free
**SF-12**	12 questions with multiple options	Scoring systems available, time consuming	5–10 min	Paper only	Multiple, with translation guidelines available	No	Yes	Free
**SOMS**	8 domains, No. of questions N/A	N/A	N/A	N/A	N/A	Partial assessment	Uncertain	N/A
**PROMIS-10**	10 questions with multiple options	Information on scoring available online	N/A	On paper, by computer, on an app	English and Spanish	No	Yes	Free in English and Spanish, payment required for other languages
**GIQLI**	36 questions with 5 options	Available online, time consuming	15–20 min	Paper only	N/A	Yes	Yes	Free

PROM, patient-reported outcome measure; SF-36^®^, Short Form 36; SF-12, short form-12; WHOQoL-BREF, World Health Organization quality of life-BREF; GIQLI, gastrointestinal quality of life index; PROMIS-10, Patient reported outcome measurement information system 10; SOMS, surgical outcomes measurement system; EQ, euro quality of life; VAS, visual analogue scale; N/A, not available.

## Discussion

This systematic review identified more than 20 relevant publications in 19 countries dealing with LC outcomes. The focus was on comprehensive PROMs and this study is different from previous reviews^39,40,10^ as information is given on each of the PROM tools identified and the parameters they measure.

Mucek and colleagues used the International Society of QoL (ISOQoL) reporting standard for PROMs, primarily health-related QoL (HRQoL) in RCTs^39^. This checklist has been available since 2013, yet despite the availability of this tool, a minority of RCTs were considered to include high-quality PRO reporting. Daliya and co-workers analysed all clinical trials evaluating HRQoL following LC^40^. They were unable to make a recommendation on PRO instruments. Alexander and colleagues aimed to determine the frequency and consistency with which PROs are measured and reported in patients undergoing LC. As with previous reviews, the present review identified significant heterogeneity in the PROM tools utilized in the studies. Alexander and colleagues identified that PRO questionnaires evaluated a wide array of concepts other than HRQoL, such as cosmesis, pain, and satisfaction^10^. The present study also found that there was significant variation in both the comprehensive HRQoL PROM used and other selective PROMs evaluated in conjunction with HRQoL. The variation in PROM selection highlights the lack of recommendations for PROMs in LC. It is important to standardize outcome reporting as LC is a very common surgical intervention worldwide. Surgeons have accepted that QoL and long-term outcomes after surgery are as important as traditional and short-term outcomes. Enhanced recovery after surgery (ERAS) protocols that involve patients for the purpose of improved long-term outcomes are beginning to be implemented in clinical practice and in patients undergoing LC^41^. Thus, it is important to have a standardized tool to measure the long-term patient outcomes and QoL in patients undergoing LC.

There are many options when it comes to choosing a PROM and the variety suggests that none is ideal for LC. Regarding ease of use, EQ-5D™ has many advantages with five questions and five optional answers on health-related issues, taking less than 1 min to complete. In addition, it is available in many formats: laptop, paper, tablet, and phone app, and is available in 200 languages. Further, there is a youth/adolescent version. It is free for non-profit use and is validated, reliable, and responsive^42^. It has a disadvantage of not assessing for diarrhoea, which is a potentially important outcome after LC.

The WHOQoL-BREF is a freely available questionnaire that contains a total of 26 questions with five possible answers on health-related issues and includes a question on mental health. The WHO has provided a guide on scoring. There are several disadvantages to using this PROM tool: it is only available in 19 different languages, and it is only available on paper^43^. The presence of a language barrier and difficulty in accessing a web-based PROM platform were two important barriers identified by Amini for implementing PROMs in clinical care^44^. Processing the data and calculating a QoL score may be an additional burden on the healthcare provider, which poses another barrier to the use of this tool. Another disadvantage is that it does not assess for diarrhoea.

The SF-36^®^ is a validated, free to use, QoL measure (available at https://www.rand.org). It has the advantage of being available in multiple languages and RAND supply translation guidelines for translating the survey into another language^45^. It takes approximately 15–20 min to complete. Scoring systems are available, which may be burdensome for the healthcare provider as the questionnaire is only available on paper. RAND-36 and SF-36^®^ are essentially the same instrument as they contain the same set of questions; however, the scoring scales differ slightly in the domains of general health and bodily pain. The time required to complete and score the SF-36^®^ may be a barrier to its implementation, along with the lack of availability of a web-based platform^44^. It also lacks a specific question on diarrhoea, which has been identified in patients following LC^46^. The SF-12 is a shortened version of the SF-36^®^ (available at https://www.rand.org)^45^. It has the same barriers to use as the SF-36^®^, but the fact that is a shortened version reduces the time burden.

SOMS is an extension of the National Institutes of Health (NIH)-funded PROMIS^35^. There is a lack of easily accessible information on SOMS online and it is unclear whether the tool is validated as Leung has described it as a validated tool, whereas Vigneswaran has said that it is not validated^35,47^. The lack of easily available information on SOMS is a significant barrier to its implementation in clinical practice.

The PROMIS-10 is a validated PROM tool. PDF versions of the PROMIS-10 are free and readily available at https://www.healthmeasures.net/explore-measurement-systems/promis/obtain-administer-measures^48^. It has the advantage that it can be administered in three ways: on paper, by computer, or with an app. It can be used for free in English and Spanish; however, other languages are subject to a distribution fee, which may be a barrier to its use globally. PROMIS-10 questions are based on the SF-36^®^ and EQ-5D™ and answers from the PROMIS-10 can be used to calculate an EQ-5D™ index score. Further information on scoring PROMIS-10 is available at https://www.codetechnology.com/promis-global-10/^49^. One problem with the PROMIS-10 for use in LC, as with the other tools, is that it does not ask about diarrhoea.

The GIQLI survey score ranges from 0 to 144 with a higher number indicating a better QoL^50^. It has the advantage of including questions on specific gastrointestinal symptoms such as diarrhoea and has been recommended for use in patients undergoing LC by the European Association for Endoscopic Surgery^18^. It has disadvantages; it is only available in paper version, and the 36 questions take 15 to 20 min to complete. Adding up the scores is also a burden on the healthcare provider. Time burden and a lack of web-based platform are barriers that Amini identified to implementing a PROM tool in clinical practice^44^.

In all studies included in this systematic review PROMs were completed either by the patients themselves, with the aid of a nurse, or with the aid of a dedicated researcher. The surveys were distributed in person, by mail, over the telephone, or by e-mail via traditional paper surveys. None of the studies used electronic or digital PROMs, despite recent advancements in technology and the availability of these modalities. Many alternatives to the traditional paper surveys are now available such as web-based platforms, laptop versions, tablet versions, and phone apps^51^. Modern methods have several advantages over the traditional paper-and-pencil method, such as being interactive and practical, minimizing data entry errors, and providing immediate scoring feedback. Despite this, there are also significant disadvantages, particularly the cost associated with licensing, potential patient discomfort with technology as these patients may require training to use the technology, and the potential for security breaches related to data transfer, computer errors, or unauthorized access to patient-reported data^51^.

Parkin and colleagues set out to establish which factors are most important to patients admitted with emergency gallstone pathology with a 41-item survey^7^. Their study highlighted that patients value long-term QoL following cholecystectomy as the most important outcome, with return to normal diet the next most important outcome. Some of the lowest-rated factors by the patients included being treated as a day case, operation duration, short-time to return to normal diet, and cosmesis. In a similar study Mak and co-workers aimed to study PROM in patients undergoing elective LC^8^. They designed a PROM questionnaire to gather information on what patients perceived to be important aspects of the surgical procedure and recovery process, their perceptions on hospital experience, and long-term outcomes. The results of their survey were in line with Parkin and colleagues. In terms of patient experience, communication skill of the surgeon and patients’ involvement in decision-making were highly ranked. Regarding long-term outcomes, patients perceived QoL to be most important, whereas scar, and cosmesis were ranked as least important, except in younger females who perceived cosmesis as important. These studies are important to consider in choosing the ideal PROM tool for use in LC. Despite the availability of these studies, and the guidelines suggesting that PROMs should be used to identify long-term outcomes that are important to patients^15^, none of the studies identified by this systematic review selected the PROM tool in consultation with patients to choose a tool that would measure long-term outcomes that were important to the patient.

Patient-reported experience measures (PREMs) measure the patient’s perception of the services provided. They are usually anonymous; however, the patient may identify themselves if they choose^52^. PREMs are a measure of patient care, and in contrast with PROMs they do not look at patient outcomes, but the impact of the care provided on the patient’s experience. PREMs can be relational, such as whether the patient felt listened to, or functional, such as, the facilities available^52^. A positive correlation has been demonstrated between the outcomes of PROMs and PREMs; patient outcomes have been shown to improve patient experiences by 10 per cent, and improved patient experiences correlate to an improvement of 3 per cent on patient outcomes in patients undergoing elective surgery^53^. PROMs cover information belonging to categories for QoL, individual care, and community, whereas PREMs provide information on service provided, provider culture, and innovation^52^. Black and co-workers’ study indicates that patients make a clear distinction between the different domains measured in PROMs and PREMs and highlights that PROMs and PREMs should be used in conjunction to gain a clear perspective on the quality of the health services provided, health outcomes, and patient experience to improve patient-centred care^53^. The current search did not identify any studies conducted that utilized both PROMs and PREMs to measure patient outcomes following LC. Using PROMs in conjunction with PREMs may provide a holistic insight into the outcome of patients undergoing LC. The lack of studies utilizing both types of tools highlights a clear and obvious gap in the literature.

There are several limitations to the present study. The search was limited to studies published between 2011 and 2021 and this may have prevented the identification of studies validating the use of PROMs in LC. Daliya and colleagues identified six PROM validation studies. These were all published before 2011 and so were not included in the current search^39^. This study utilized articles published only in the English language, which may have prevented the identification of some PROM tools used in LC. Four gallstone-disease-specific PROMs have been developed^54^. The present search did not identify any gallstone-specific PROMs; however, these do not seem to have gained traction in LC as they have not been utilized in the recent literature.

Studies reporting the use of PROMs in transvaginal LC patients, paediatric patients, and pregnant patients undergoing LC were not included to maintain heterogeneity in the study. The ideal choice of PROM tool for use in these groups may differ from the cohort of patients undergoing LC included here.

In terms of ease of use, minimal time burden, availability on multiple platforms, reliability, and responsiveness, and being easy to interpret, the EuroQoL EQ-5D™ has many of the desirable factors outlined in Amini’s paper on facilitators and barriers for implementing PROMs in clinical care^44^. It is also a long-term measure of QoL that has been identified by Parkin and Mak as one of the most important factors to patients undergoing LC^7,8^. Diarrhoea is one symptom that has been identified in post-cholecystectomy syndrome and is a significant burden to patients^13,45^. A modified version of the EQ-5D™ to include questions evaluating gastrointestinal symptoms may make it the most suitable of the PROM tools identified in this study for use in patients undergoing LC. This would involve using the existing EQ-5D™ as a template and adding relevant questions investigating gastrointestinal symptoms such as diarrhoea; a new scoring system would also have to be developed. The fact that this PROM tool requires modification, and the heterogeneity in selection of PROMs in studies on patients undergoing LC suggests that there is no currently available PROM that is ideal for LC. Future studies may aim to develop a new PROM specifically for use in LC. The PROM tool should be administered before and after surgery in future studies; the most important scores generated by PROMs are usually the changes from before to after an intervention^11^. LC is a procedure that is often performed to improve QoL, so it is important to investigate whether patients are better off after the procedure^38^. Only two-thirds of the studies included in this systematic review administered the PROM tool both before and after surgery, highlighting the variation in PROM utilization and reporting. Also indicating that trials are not adhering to the established comprehensive guidelines as described in the CONSORT PRO extension, where it is recommended that baseline PROM data should be collected and reported^55^.

This study identified an array of PROMs that have been used after LC. PROMs are currently infrequently measured and may not capture all outcomes but should be incorporated into future biliary and cholecystectomy research. EuroQoL EQ-5D™ provides a simple platform for the modern digital era.

## Supplementary Material

zrac062_Supplementary_Data

## Data Availability

All supporting data are included within the main article and its supplementary files.
